# ﻿Five new species in the genus *Staurosirella* (Bacillariophyta) from European freshwater habitats

**DOI:** 10.3897/phytokeys.242.122458

**Published:** 2024-05-31

**Authors:** Bart Van de Vijver, Valérie Peeters, Iris Hansen, Petra Ballings, Myriam de Haan

**Affiliations:** 1 Meise Botanic Garden, Research Department, Nieuwelaan 38, 1860 Meise, Belgium Meise Botanic Garden Meise Belgium; 2 University of Antwerp, Department of Biology – ECOSPHERE, Universiteitsplein 1, 2610 Wilrijk, Belgium University of Antwerp Wilrijk Belgium; 3 Direction régionale Bourgogne-Franche-Comté, Service Connaissance, Office français de la biodiversité, 57 rue de Mulhouse 21000 Dijon, France Direction régionale Bourgogne-Franche-Comté, Service Connaissance, Office français de la biodiversité Dijon France; 4 Marine and Freshwater Research Institute, Fornubúðir 5, 220 Hafnarfjörður, Iceland Marine and Freshwater Research Institute Hafnarfjörður Iceland

**Keywords:** Europe, morphology, new species, Staurosirella, taxonomy

## Abstract

Several populations belonging to the genus *Staurosirella* have been observed in European rivers that were previously identified as *Staurosirellapinnata*. In light of the recent taxonomic revisions of the genus *Staurosirella*, the morphology of the unknown *Staurosirella* populations has been critically investigated using light and scanning electron microscopy. Following the comparison with previously described *Staurosirella* species, five taxa could not be identified using the currently available literature on the genus. These five taxa are described as new based on differences in valve outline; shape, size and structure of the apical pore fields; structure of the striae; and the presence, position and structure of the marginal spines. Two new species were described using historic collection material: *Staurosirellabinodiformis***sp. nov.** and *Svanheurckiana***sp. nov.** Two new species were observed in samples from rivers in Flanders: *S.marginostriata***sp. nov.** and *S.stoksiana***sp. nov.** whereas a fifth species was observed in rivers from Iceland: *S.jonssoniana***sp. nov.** All new species are compared with similar *Staurosirella* species worldwide. Notes are added on their ecological preferences derived from both physicochemical data and the associated diatom flora.

## ﻿Introduction

The genus *Staurosirella* D.M.Williams & Round was split in 1988 from the genus *Fragilaria* Lyngbye sensu lato (Williams and Round 1988) and is characterised by both isopolar or heteropolar valves with broad uniseriate striae composed of slit-like, linear, internally occluded areolae separated by narrow vimines. The original 1988 description was refined in 2006 (Morales and Manoylov 2006a). Since its erection, more than 50 *Staurosirella* species were either described as new species or transferred to it from other genera such as *Fragilaria*, *Odontidium* Kützing, *Staurosira* Ehrenberg or *Opephora* P.Petit (e.g. Morales and Manoylov 2006b; Van de Vijver et al. 2014; Almeida et al. 2015; Seeligmann et al. 2018; Guerrero et al. 2019; Morales et al. 2019, Osório et al. 2021; Van de Vijver 2022, 2023, Van de Vijver et al. 2022).

In the past 10 years, the number of *Staurosirella* species strongly increased, most likely due to a better understanding of several catch-all species such as *Staurosirellapinnata* (Ehrenberg) D.M.Williams & Round. An internet search for the name ‘*Staurosirellapinnata*’ resulted in more than 12.000 hits with an extra 38.700 for the name ‘*Fragilariapinnata*’. For comparison, the name ‘*Staurosirellaneopinnata* E.Morales et al.’ only resulted in 907 hits. Moreover, the name ‘Staurosirella (Fragilaria) pinnata’ was reported worldwide from the tropics to the poles with more than 80 records from Asia, Africa, Antarctica, Australia, North, Central and South America, and Europe [see AlgaeBase] (Guiry and Guiry 2023). It is clear that this worldwide distribution is the result of taxonomic drift on one side (severely broadening the original description of the species) and force-fitting (Tyler 1996) populations from all over the world into *S.pinnata*. Most of the confusion was linked to a lack of knowledge of the type material of *S.pinnata* as can be seen in Krammer and Lange-Bertalot (1991, plate 133) that shows the complex of species related to *Fragilariapinnata* and *F.leptostauron*. The former proved to belong to the genus *Denticula* (Morales et al. 2013) resulting in the description of a new species, *S.neopinnata* to accommodate some of the populations, formerly identified as *S.pinnata*. Since our knowledge of the morphology and taxonomic identity of this seemingly widespread species improved significantly, a lot of species, previously included within *S.pinnata*, have been described, several of them from Europe, such as *Staurosirellacoutelasiana* Van de Vijver, *S.minutissima* Van de Vijver, *S.eruciformis* Van de Vijver and *S.lucectoriana* Beauger, C.E.Wetzel & Van de Vijver (Van de Vijver 2022, 2023; Beauger et al. 2023).

During a survey of *Staurosirella* populations in European rivers in the framework of a routine water quality biomonitoring exercise, a large number of unknown taxa has been recorded that formerly were identified as *S.pinnata* s.l. Detailed morphological analysis of the different populations indicated several morphological differences with the type population of *S.neopinnata* (as the species should be called since 2019). Comparison with all available (recent) literature, exposed that several of these populations showed sufficient morphological differences to justify their description as new species despite a growing number of described *Staurosirella* species from all continents (see Material and Methods for a complete overview of all used literature). The present contribution describes five new species based on detailed light (LM) and scanning electron (SEM) microscopy and comparisons with all known taxa worldwide: *Staurosirellabinodiformis* Van de Vijver, sp. nov., *S.marginostriata* Van de Vijver & V.Peeters, sp. nov., *S.stoksiana* Van de Vijver, sp. nov., *S.jonssoniana* Van de Vijver & Iris Hansen, sp. nov., and *S.vanheurckiana* Van de Vijver, Ballings & M.de Haan, sp. nov. Information on their ecological preferences is derived from the associated diatom flora and, when available, measured physico-chemical parameters.

## ﻿Material and methods

In the present paper, a mixture of historic (herbarium) materials and recently collected samples have been investigated to detail the morphological features of the new *Staurosirella* species. The following samples have been used in this investigation:

- Sample APM21-91, Bosbeek (Maaseik, Province of Limburg, Belgium), leg. Vlaamse Milieu maatschappij (VMM), 51°5.6348'N, 5°45.894'E, coll. date 25 Jun. 2021.
- Sample Foged 29/1954, outflow from a small lake near Þingvellir, Iceland, coll. date 15 Jul. 1954, leg. Niels Foged (original material kept in C!)
- Sample 81a, Voorste Nete (Dessel, Province of Antwerp, Belgium), 51°13.9482'N, 5°7.4497'E, coll. date 06 Jul. 1994, leg. B. Van de Vijver.
- Sampling site at Græntorfa, Grenlækur, southern, Iceland, 63°43.955'N, 17° 58.067'W, coll. date 03 Jul. 2017, leg. Iris Hansen.
- Types du Synopsis sample 315, Leuven, Belgium, Van Heurck exsiccata set, leg. (probably) Père Gautier

A sub-sample of each of the selected materials was prepared for LM and SEM observations following the method described in van der Werff (1955). Small parts of the sub-samples were cleaned by adding 37% H_2_O_2_ and heating to 80 °C for about 1 h, after which the reaction was completed by addition of saturated KMnO_4_. Following digestion and centrifugation (three times 10 minutes at 3700× rpm), the resulting cleaned diatom material was diluted with distilled water to avoid excessive concentrations of diatom valves on the slide and mounted on permanent slide using Naphrax (refraction index 1.73). The resulting slides were analysed using an Olympus BX53 microscope at 1000× magnification (UPlan FL N 100× oil objective, N.A. 1.30), equipped with Differential Interference Contrast (Nomarski) optics and the Olympus UC30 Imaging System, connected to the Cell Sense Standard program. For each taxon, the number of specimens, measured at random on the type slide, is indicated (n=X). For each species, at least 15, but often many more, valves are illustrated using LM to determine its morphological variability. An ecological characterisation of the new species is added based on the accompanying diatom flora, assessed by counting at least 100 diatom valves along random transects. Relative abundances, when given, are expressed as percentage of counted valves.

For SEM, part of the suspension was filtered through 5-μm Isopore™ polycarbonate membrane filters (Merck Millipore), pieces of which were fixed on aluminum stubs after air–drying and coated with a platinum layer of 20 nm, and studied using a JEOL-JSM-7100F field emission scanning electron microscope at 2 kV. Slides, samples and stubs are stored at the BR-collection (Meise Botanic Garden, Belgium). Plates were prepared using Photoshop CS5.

Terminology used in the description of the various structures of the siliceous cell wall is based on Ross et al. (1979, areola structure), Cox and Ross (1981, stria structure), Morales (2005, girdle structure), Williams and Round (1988, *Staurosirella* genus features) and Morales and Manoylov (2006a,b, *Staurosirella* genus features). For taxonomic comparisons, the following papers were consulted: Lange-Bertalot (1993), Morales (2005), Morales and Manoylov (2006a, 2006b), Morales et al. (2010a, 2010b, 2015, 2019), Almeida et al. (2015), Guerrero et al. (2019), Osório et al. (2021), Seeligmann et al. (2018), Van de Vijver et al. (2014), Van de Vijver (2022, 2023), and Beauger et al. (2023).

For typification of the species, we chose to use the entire slide as the type, following article 8.2 of the International Code of Nomenclature for algae, fungi, and plants (Turland et al. 2018). Diatoms show a broad variability along their cell cycle making the choice for the entire population on the slide more obvious, but because of admixtures, one valve was indicated to best illustrate the taxon (see Figures). All novelties are registered proactively according to Art. 42.3 (Turland et al. 2018).

## ﻿Results

### ﻿ Staurosirella marginostriata

Taxon classificationPlantaeFragilarialesStaurosiraceae

Van de Vijver & V.Peeters sp. nov.

6EBB8AF9-6DA2-5E0C-ACC1-02AF71F25D51

Fig. 1

#### Holotype.

**Figure 1.**
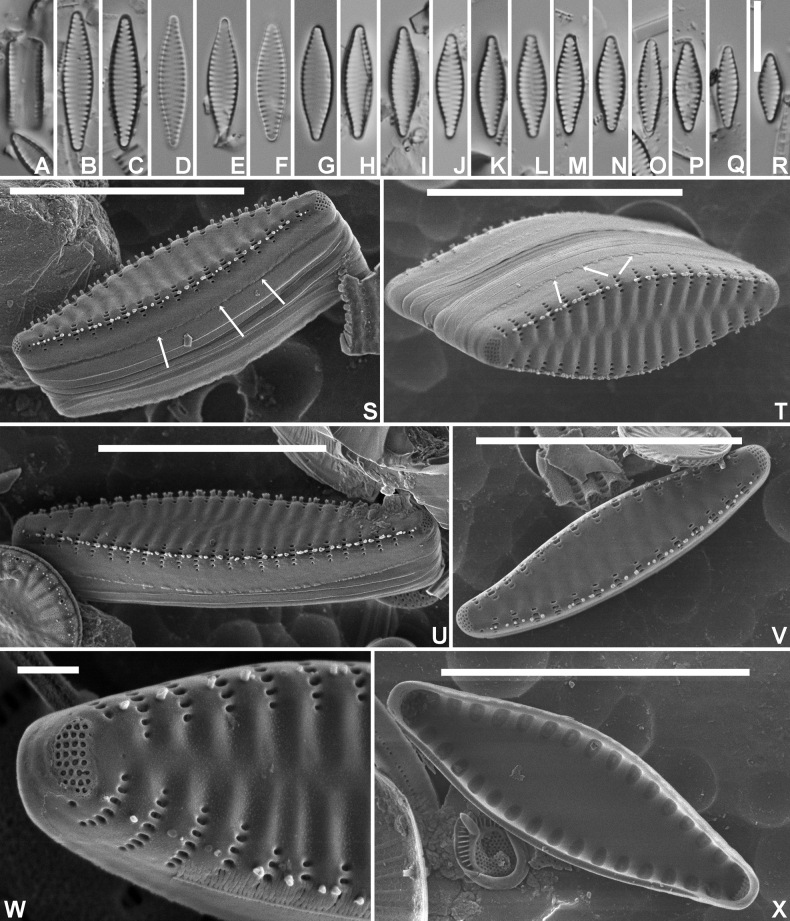
*Staurosirellamarginostriata* Van de Vijver & V.Peeters, sp. nov., LM and SEM micrographs taken from the holotype material (BR-4840, Bosbeek, Maaseik, Belgium) **A** LM picture of a frustule in girdle view **B**–**R** LM pictures of valves in valve face view in decreasing length **S**SEM external view of a complete valve in oblique view showing the girdle structure and the mantle **T**SEM external view of a complete frustule with focus on the apical pore field and the transition between valve face and mantle **U**SEM external view of a complete valve. Note the undulating valve face surface and the small mantle plaques (see arrows) **V**SEM external view of a complete smaller valve with flattened valve face surface. The arrows indicate mantle plaques **W**SEM external detail of a valve apex showing the large apical pore field **X**SEM internal view of a complete valve. Scale bars: 10 µm (**A–V, X**); 1 µm (**W**).

BR-4840 (Meise Botanic Garden, Belgium). Fig. 1C represents the holotype.

#### Isotype.

Slide 442 (University of Antwerp, Belgium).

#### Registration.

http://phycobank.org/104533.

#### Type locality.

Bosbeek (Maaseik, Province of Limburg, Belgium), sample APM21-91, 51°5.6348'N, 5°45.894'E, coll. date 25 Jun. 2021, leg. Vlaamse Milieu maatschappij (VMM).

#### Description.

***LM*** (Fig. 1A–R). Frustules in girdle view rectangular, singular. Chains linking several cells in colonies so far not observed. Valves isopolar, strictly lanceolate with convex margins and weakly protracted, rostrate apices. Smaller valves more rhombic-lanceolate with cuneate apices (Fig. 1R). Valve dimensions (n=20): length 10–20 µm, width 3.0–4.5 µm. Sternum broad, lanceolate, ghost striae present. Striae short, marginal, 14–15 in 10 µm. Areolae not discernible in LM. ***SEM*** (Fig. 1S–X). Valve face surface undulating with distinctly raised virgae extending almost up to the valve middle (Fig. 1T), occasionally almost flat (Fig. 1V). Virgae much broader that the striae. Striae very short, marginal, extending without interruption from the valve face onto the mantle (Fig. 1S–U, W). Large hyaline zone present at the abvalvar mantle edge (Fig. 1S–U), terminating at mantle edge by series of irregularly shaped mantle plaques (Fig. 1S–U). Striae uniseriate, composed of short, slit-like, linear areolae, running parallel to the apical axis (Fig. 1S–W). Vimines narrow, not raised. Marginal spines located on the virgae, irregular in number (1–3), shape and size (Fig. 1S–W). Large apical pore fields present at both apices, usually similar in size and shape (Fig. 1S, T, V), located at the valve face/mantle junction, extending more onto the valve mantle, isolated from neighboring striae. Pore fields composed of usually 4–5 rows of small, rimmed pores (Fig. 1W). Girdle composed of several probably open, plain copulae (Fig. 1S–U). Internally, striae distinctly sunken between the raised virgae and the sternum (Fig. 1X).

#### Etymology.

The specific epithet *marginostriata* refers to the short, marginal striae.

#### Distribution.

At present, only observed in Flanders (type locality) and the Morvan region in France. Confusion with the in LM similarly looking *Pseudostaurosirabrevistriata* (Grunow) D.M.Williams & Round), may be at the base of the unclear distribution.

#### Ecology and associated diatom flora.

The type locality has an almost circumneutral pH (6.9–7.3), moderate conductivity (160–200 µS/cm), higher nitrate levels (1.4–3.3 mg/l) and sulphate levels (12–30 mg/l). The sample is dominated by *Staurosirellastoksiana* Van de Vijver sp. nov. (15% of all counted valves), *Aulacoseiraambigua* (Grunow) Simonsen (12.5%), *Pseudostaurosirabrevistriata* (9.5%), *A.granulata* (Grunow) Simonsen (9%), *Naviculacryptocephala* Kützing (5%), and *N.lanceolata* (C.Agardh) Ehrenberg (4%), pointing to more meso-eutrophic, alkaline conditions (Lange-Bertalot et al. 2017).

### ﻿ Staurosirella binodiformis

Taxon classificationPlantaeFragilarialesStaurosiraceae

Van de Vijver sp. nov.

6A343AFD-5CFB-5BA7-A5CF-E58BBD943C9A

Fig. 2

#### Holotype.

**Figure 2.**
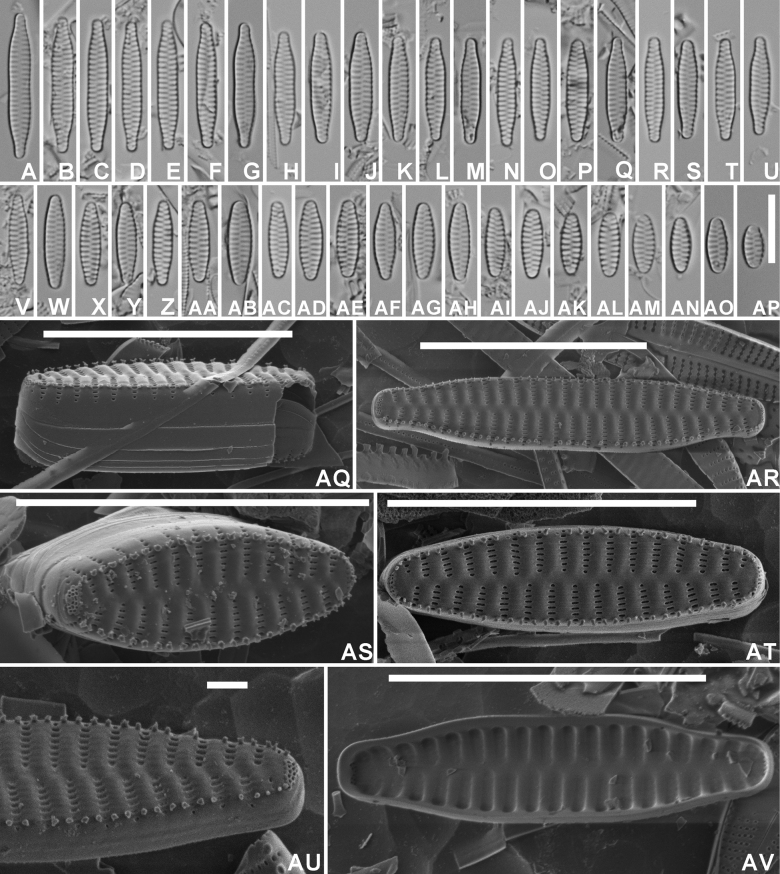
*Staurosirellabinodiformis* Van de Vijver, sp. nov., LM and SEM micrographs taken from the holotype material (BR-4841, Foged sample 29) **A**–**AP** LM pictures of valves in valve face view in decreasing length **AQ**SEM external view of a complete valve in girdle view showing the girdle structure and the mantle **AR**SEM external view of a complete valve. Note the slight constriction at the valve middle, the undulating valve face surface and the series of spines **AS**SEM external view of a complete valve with focus on the apical pore field and the transition between valve face and mantle **AT**SEM external view of a complete valve **AU**SEM external detail of a valve apex showing the large apical pore field **AV**SEM internal view of a complete valve. Scale bars: 10 µm (**A–AS, AV**); 1 µm (**AU**).

BR-4841 (Meise Botanic Garden, Belgium). Fig. 2E represents the holotype.

#### Isotype.

Slide 443 (University of Antwerp, Belgium).

#### Registration.

http://phycobank.org/104534.

#### Type locality.

outflow from a small lake near Þingvellir, Iceland, sample 29, coll. date 15 Jul. 1954, leg. Niels Foged.

#### Description.

***LM*** (Fig. 2A–AP). Valves isopolar, linear, slightly constricted near the valve center in longer specimens (Fig. 2A–G), becoming linear, linear-lanceolate to even almost elliptical in smallest specimens (Fig. 2AM–AP). Apices in longer specimens protracted, rostrate to subcapitate, to not protracted, broadly rounded in smaller valves. Valve dimensions (n=50): length 6–21 µm, width 3.0–3.5 µm. Sternum narrow, linear. Striae almost parallel weakly radiate in the valve center, becoming more radiate towards the apices, long, almost reaching the sternum, 14–15 in 10 µm. Areolae not discernible in LM. ***SEM*** (Fig. 2AQ–AV). Valve face surface weakly undulating with raised virgae extending almost up to the valve middle (Fig. 2AR–AT). Virgae almost double as broad as the striae. Striae long, extending without interruption from the valve face onto the mantle (Fig. 2AQ). Very large hyaline zone present at the abvalvar mantle edge (Fig. 2AQ). Mantle plaques absent (Fig. 2AQ). Striae uniseriate, composed of slit-like, linear areolae, running parallel to the apical axis (Fig. 2AQ–AU). Areolae diminishing in size towards the sternum (Fig. 2AT), becoming almost rounded at the sternum. Mantle striae often very short, reduced to only one areola (Fig. 2AU), occasionally also longer (Fig. 2AS). Vimines very narrow, not raised. Irregular but dense series of short, marginal spines located on the virgae, usually bordering immediately the striae (Fig. 2AR–AS). Spines occasionally bifurcated (Fig. 2AQ, AS). Apical pore fields present at both apices, usually similar in size and shape (Fig. 2AR–AT), located at the valve face/mantle junction, extending more onto the valve mantle, isolated from neighbouring striae. Pore fields composed of usually 2–3 rows of small, rimmed pores (Fig. 2AU). Girdle composed of several open, plain copulae (Fig. 2AQ). Internally, striae distinctly sunken between the raised virgae and the sternum (Fig. 2AV).

#### Etymology.

The specific epithet *binodiformis* refers to the outline resemblance with *Staurosirabinodis* Ehrenberg.

#### Distribution.

At present, only observed in Iceland on the type locality, probably due to confusion with *Staurosirabinodis* and *Staurosirellaoldenburgiana* (Hustedt) E.Morales.

#### Ecology and associated diatom flora.

Niels Foged (1906–1988) collected the sample in 1954 from stones covered with moss and green algae in the outflow from a small lake near Þingvellir on the road between Reykjavik and Þingvellir, north of Mosfellsheidi (Iceland). The sample is dominated by a large number of small-celled araphid taxa belonging to the genera *Staurosira*, *Staurosirella* and *Pseudostaurosira* D.M.Williams & Round. As most of these taxa most likely belong to currently undescribed species, it is hard to derive the ecology from them. Several raphid taxa were observed, but in much lower frequencies such as *Placoneisexplanata* (Hustedt) Mayama, *Planothidiumjoursacence* (Héribaud) Lange-Bertalot, *Skabitschewskiaperagallii* (Brun & Héribaud) Kulikovskiy & Lange-Bertalot, *Cavinulajaernefeltii* (Hustedt) D.G.Mann & Stickle, and *C.pusio* (Cleve) Lange-Bertalot. Most likely, the diatom flora points to oligo- to mesotrophic, colder, pioneer conditions (Lange-Bertalot et al. 2017).

### ﻿ Staurosirella stoksiana

Taxon classificationPlantaeFragilarialesStaurosiraceae

Van de Vijver sp. nov.

10BEA553-050B-5BEE-8338-A2812D283A9C

Figs 3 , 4

#### Holotype.

**Figure 3.**
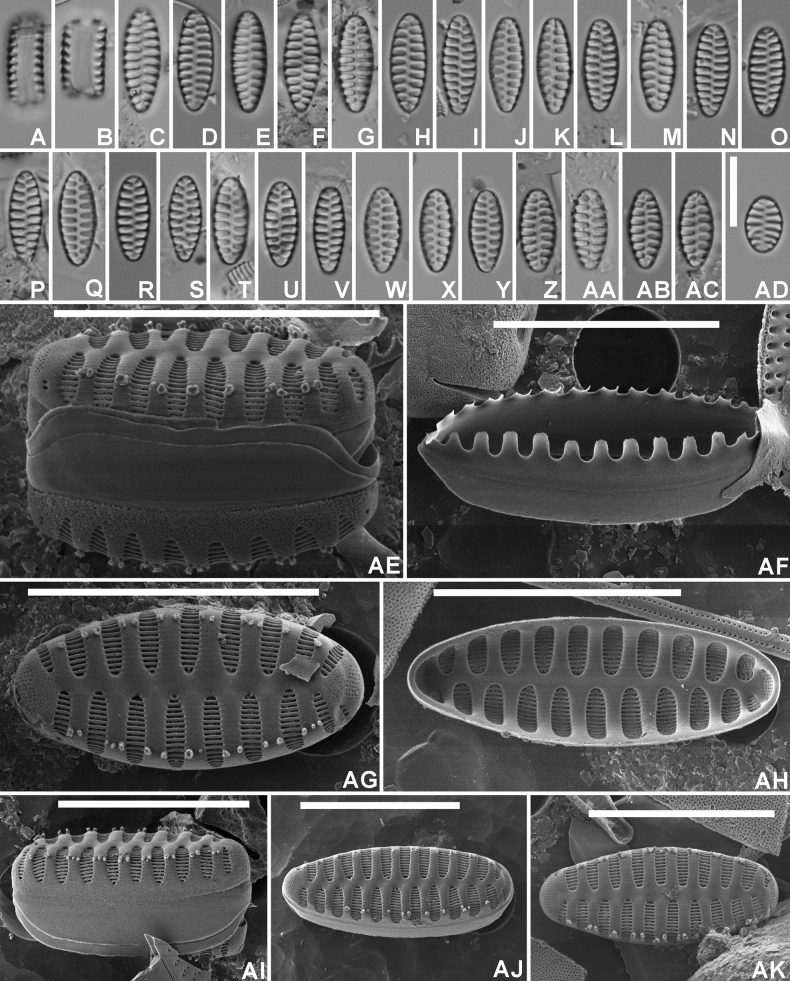
*Staurosirellastoksiana* Van de Vijver, sp. nov., LM and SEM micrographs taken from the holotype material (BR-4842, Voorste Nete, Dessel, Belgium) **A, B** LM pictures of a frustule in girdle view **C**–**AD** LM pictures of valves in valve face view in decreasing length **AE**SEM external view of a complete frustule in girdle view showing the girdle structure and the mantle **AF** Complete view of the valvocopula with the large fimbriae **AG**SEM external view of a complete valve with focus on the apical pore field and the depressed headpole **AH**SEM internal view of a complete valve **AI**SEM external view of a complete valve in girdle view **AJ–AK** Two SEM externals view of a complete, heteropolar valve. Scale bars: 10 µm.

BR-4842 (Meise Botanic Garden, Belgium). Fig. 3H represents the holotype.

#### Isotype.

Slide 444 (University of Antwerp, Belgium).

#### Registration.

http://phycobank.org/104535.

#### Type locality.

Voorste Nete (Dessel, Province of Antwerp, Belgium), sample 81a, 51°13.9482'N, 5°7.4497'E, coll. date 06 Jul. 1994, leg. B. Van de Vijver.

#### Additional investigated population.

Bosbeek (Maaseik, Province of Limburg, Belgium), sample APM21-91, 51°5.6348'N, 5°45.894'E, coll. date 25 Jun. 2021, leg. Vlaamse Milieu maatschappij (VMM).

#### Description.

**Figure 4.**
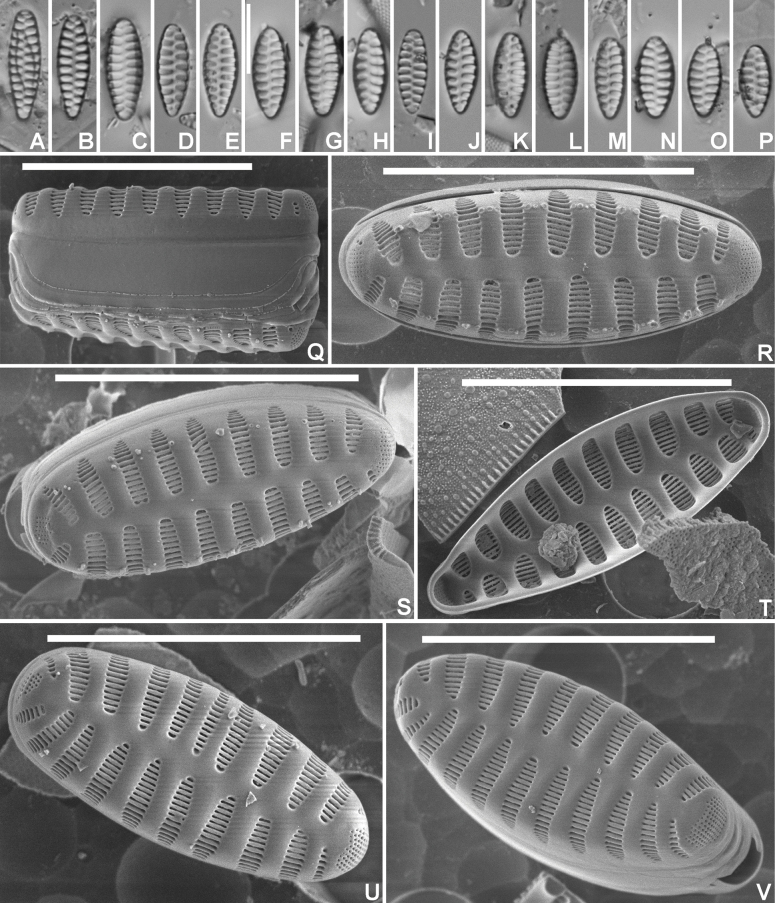
*Staurosirellastoksiana* Van de Vijver, sp. nov., LM and SEM micrographs taken from an additional population (BR-4840, Bosbeek, Maaseik, Belgium) **A–P** LM pictures of valves in valve face view in decreasing length **Q**SEM external view of a complete frustule in girdle view showing the girdle structure and the mantle **R, S**SEM external view of two complete valves with focus on the apical pore field and the depressed headpole. Both valves bear a series of marginal spines **T**SEM internal view of a complete valve **U, V** Two SEM externals view of a complete, heteropolar valve lacking marginal spines. Scale bars: 10 µm.

***LM*** (Figs 3A–AD, 4A–P). Frustules rectangular, solitary. Chain-like colonies so far not observed. Valves weakly heteropolar, lanceolate to elliptic-lanceolate with convex margins, gradually narrowing towards the non-protracted, broadly rounded apices. Larger valves occasionally slightly ovoid (Fig. 3C) in outline with smallest valves almost elliptical (Fig. 3AD). Valve dimensions (n=40): length 6–21 µm, width 4.5–5.5 µm. Sternum narrow, linear. Striae alternating, almost parallel becoming very slightly radiate towards the apices, long, almost reaching the sternum, 8–9 in 10 µm. Areolae not discernible in LM. ***SEM*** (Figs 3AE–AK, 4Q–V). External valve face undulating with slightly raised, flattened virgae and striae sunken in ‘punch hole-like’ depressions (Figs 3AE, 4Q). Striae extending without interruption from valve face onto the mantle, gradually but distinctly narrowing at both ends (Figs 3AE, AI, 4S) giving the striae a lanceolate appearance. Abvalvar mantle edge forming a broad hyaline zone, almost half the entire mantle width (Figs 3AE, 4Q). Mantle plaques absent. Striae uniseriate, composed of long, slit-like, linear areolae, running parallel to the apical axis (Figs 3AE, AG, AJ, AK, 4R, S, U, V). Vimines very thin, not raised. Marginal spines usually present, one to two, located on the virgae, rudimentary, irregularly shaped (blunt, thick, papilla-like). Occasionally mixed populations with spineless and spine valves observed (Fig. 4R, S, U, V). Apical pore fields present at both apices. At the footpole, pore field rather large, composed of at least 7 long rows of very small, rounded pores, covering the entire foot pole (Figs 3AG, 4U–V). Pore field at the headpole smaller, located on the weakly depressed headpole (Fig. 3AG, AJ, AK).Valvocopula broad, with short but well developed fimbriae (Fig. 3AF). Internally, striae distinctly sunken between the flat, doubly flared virgae and sternum (Figs 3AH, 4T). Areolae occluded by irregularly shaped volae, extending from the longer inner side of each vimen (Fig. 3AH).

#### Etymology.

The new species honours Prof. dr Robby Stoks (Catholic University of Leuven) study friend of the first author, in recognition of his important contributions to the Odonata research.

#### Distribution.

*Staurosirellastoksiana* has been regularly observed in samples from Flanders (Belgium) and the United Kingdom. Most likely the new species has a broader distribution area but due to confusion with the presumably widespread Staurosirella (neo) pinnata and other similar species such as *Staurosirellaovata* and *S.coutelasiana*, its exact distribution is not clear. Peeters and Ector (2017, p. 262–263) illustrated several populations from Burgundy (eastern France) under the name *Staurosirellaovata*, that shows a very large similarity to *S.stoksiana.*

#### Ecology and associated diatom flora.

The type population of *S.stoksiana* was observed in a small lowland river in the Netebekken (Flanders, Belgium). The sample was dominated, apart from *S.stoksiana* (16% of all counted valves) by *Nitzschiaadamata* Hustedt (8.7%), *Geissleriadecussis* (Østrup) Lange-Bertalot & Metzeltin (6.4%), *Naviculagregaria* Donkin (6%), *Craticulamolestiformis* (Hustedt) Mayama (5.1%), *Melosiravarians* C.Agardh (3.4%), *Planothidiumfrequentissimum* (Lange-Bertalot) Lange-Bertalot (3.2%), and *Cyclotellameneghiniana* Kützing (3.2%). According to the ecological preferences of the observed diatom flora, this indicates meso- to eutrophic, alkaline conditions with medium conductivities (Lange-Bertalot et al. 2017).

### ﻿ Staurosirella jonssoniana

Taxon classificationPlantaeFragilarialesStaurosiraceae

Van de Vijver & Iris Hansen sp. nov.

919F6EC7-C0C7-556C-A07B-66D70417FE17

Fig. 5

#### Holotype.

**Figure 5.**
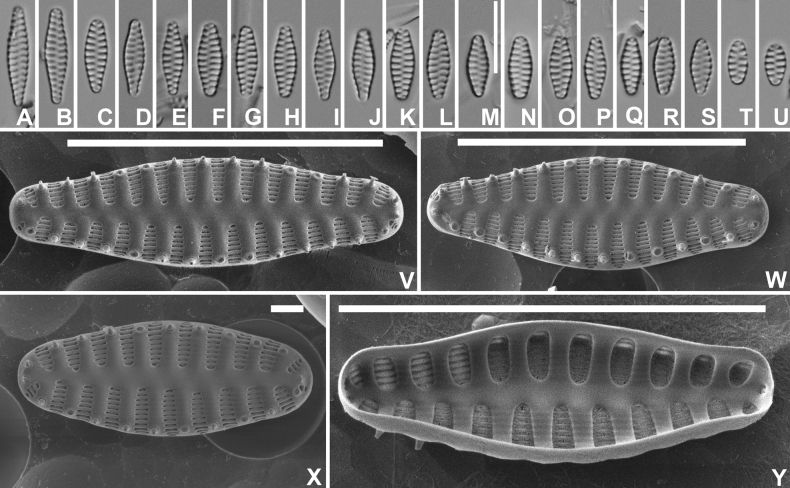
*Staurosirellajonssoniana* Van de Vijver & Iris Hansen, sp. nov., LM and SEM micrographs taken from the holotype material (BR-4843, Grenlækur, southern Iceland) **A**–**U** LM pictures of valves in valve face view in decreasing length **V–X**SEM external view of three complete valves with focus on the spines located in pit-like depressions. Note the absence of apical porefields **Y**SEM internal view of a complete valve. Scale bars: 10 µm (**A–W, Y**); 1 µm (**X**).

BR-4843 (Meise Botanic Garden, Belgium). Fig. 5H represents the holotype.

#### Isotype.

Slide 445 (University of Antwerp, Belgium).

#### Registration.

http://phycobank.org/104536.

#### Type locality.

Grenlækur, southern Iceland, sampling site at Græntorfa, 63°43.96'N, 17° 58.07'W, coll. date 03 Jul. 2017, leg. Iris Hansen.

#### Description.

***LM*** (Fig. 5A–U). Valves isopolar to weakly heteropolar, lanceolate with convex margins, and narrowly protracted, rostrate apices. Smallest valves almost elliptical (Fig. 5T–U). Valve dimensions (n=25): length 6–15 µm, width 3.0–3.5 µm. Sternum narrow, linear. Striae alternating, almost parallel to very slightly radiate in the middle, more strongly radiate towards the apices, 12–13 in 10 µm. Areolae not discernible in LM. ***SEM*** (Fig. 5V–Y). External valve face weakly undulating with slightly raised virgae and striae sunken in ‘punch hole-like’ depressions (Fig. 5V–X). Striae extending without interruption from valve face onto the mantle (Fig. 5X), gradually narrowing towards the sternum (Fig. 5V–X) giving the striae a lanceolate appearance. Striae uniseriate, composed of long, slit-like, linear areolae, running parallel to the apical axis (Fig. 5V–X). Vimines very thin, not raised. Marginal spines very obvious, one per virga, each located in a shallow, pit-like depression (Fig. 5V–X). Apical pore fields absent, replaced by one or two spines. Internally, striae distinctly sunken between the flat, doubly flared virgae and sternum (Fig. 5Y). Areolae occluded by irregularly shaped volae, extending from the longer inner side of each vimen (Fig. 5Y).

#### Etymology.

The new species honours our friend and colleague Gunnar Steinn Jónsson (Reykjavik, Iceland) in recognition of his important contributions to the diatom research in Iceland.

#### Distribution.

*Staurosirellajonssoniana* has so far only been found in Iceland.

#### Ecology and associated diatom flora.

The type locality, Grenlækur, is a small spring-fed stream in southern Iceland. The stream has a slightly alkaline pH (7.8), a rather low conductivity (156 µS/cm), low nitrate (0.02 mg/l) and phosphate (0.97 mg/l), and moderate sulphate (37.8 mg/l) levels. The diatom flora in the sample is quite diverse and dominated by a large number of species with *Staurosirellajonssoniana* only being relatively rare in the sample. The dominant species include *Fragilarialandnama* Van de Vijver & Iris Hansen, *F.sandellii* Van de Vijver & Jarlman, Staurosiracf.sviridae Kulikovskiy et al., *Planothidiumlanceolatum* (Brébisson) Lange-Bertalot, different *Cocconeis* species (mainly *C.euglypta* Ehrenberg), *Surirellabrebissonii* Krammer & Lange-Bertalot, Gomphonemapumilumvar.rigidum E.Reichardt & Lange-Bertalot, although none of them reaches more than 10% of all counted valves. Rarer species include *Odontidiummesodon* (Ehrenberg) Kützing, *Navicularadiosa* Kützing, *N.slesvicensis* Grunow and *Amphoraovalis* (Kützing) Kützing. The flora points to colder, fast-flowing, meso- to eutrophic, alkaline conditions (Lange-Bertalot et al. 2017; Van de Vijver et al. 2023).

### ﻿ Staurosirella vanheurckiana

Taxon classificationPlantaeFragilarialesStaurosiraceae

Van de Vijver, Ballings & M.de Haan sp. nov.

0CF3BBAB-18CF-5122-AB62-BC821F80EC86

Fig. 6

#### Holotype.

**Figure 6.**
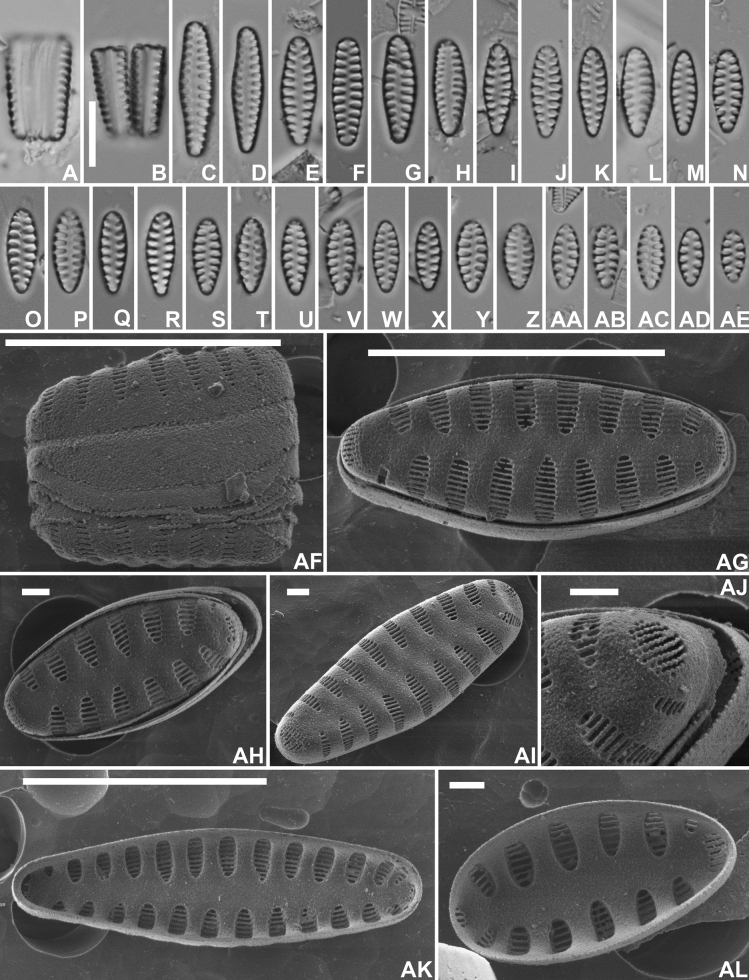
*Staurosirellavanheurckiana* Van de Vijver, Ballings & M.de Haan, sp. nov., LM and SEM micrographs taken from the holotype material (BR-4844, Van Heurck Types du Synopsis 315, Leuven, Belgium) **A, B** LM pictures of a frustule in girdle view **C–AE** LM pictures of valves in valve face view in decreasing length **AF**SEM external view of a complete (slightly eroded) frustule in oblique view showing the girdle structure and the mantle **AG–AI**SEM external view of a three complete heteropolar valves with focus on the apical pore field. Note the depressed headpole in AI **AJ**SEM external detail of a valve apex showing the large apical pore field **AK–AL**SEM internal view of two complete valve. Scale bars: 10 µm (**A–AG, AK**); 1 µm (**AH–AJ, AL**).

BR-4844 (Meise Botanic Garden, Belgium). Fig. 6J represents the holotype.

#### Isotype.

Slide 446 (University of Antwerp, Belgium).

#### Registration.

http://phycobank.org/104537.

#### Type locality.

Leuven, Belgium, Van Heurck exsiccata set Types du Synopsis 315, leg. (probably) Père Gautier

#### Description.

***LM*** (Fig. 6A–AE). Frustules in girdle view rectangular, solitary or in pairs. Ribbon-like colonies so far not observed. Valves heteropolar, lanceolate to elliptic-lanceolate throughout the entire cell diminution series, with convex margins, gradually narrowing towards the acutely rounded foot pole. Head pole broadly rounded. Smaller specimens ovoid in shape (Fig. 6S–AE). Valve dimensions (n=40): length 8–22 µm, width 3.0–3.5 µm. Sternum moderately narrow, lanceolate. Striae alternating, almost parallel to very slightly radiate throughout, 8–10 in 10 µm. Areolae not discernible in LM. ***SEM*** (Fig. 6AF–AL). External valve face almost flat with very slightly raised virgae and striae sunken in ‘punch hole-like’ depressions (Fig. 6AI). Abvalvar mantle edge forming a broad hyaline zone, almost half the entire mantle width (Fig. 6AF). Virgae broader than the striae. Striae extending without interruption from valve face onto the mantle (Fig. 6AI–AJ), gradually narrowing towards the ends (Fig. 6AF–AI) giving the striae a lanceolate appearance. Striae uniseriate, composed of long, slit-like, linear areolae, running parallel to the apical axis (Fig. 6AG–AI). Vimines very thin, not raised. Spines absent. Apical pore fields present, different on both apices. Smallest pore field present at head pole, located on small depression (Fig. 6AG–AI). At footpole, apical pore field large, composed of a large number of parallel rows of very small rounded pores (Fig. 6AJ). Internally, striae distinctly sunken between the flat, doubly flared virgae and sternum (Fig. 6AK–AL). Due to erosion, areola occlusions no longer observed.

#### Etymology.

The new species is named in honour of Henri Van Heurck (1838–1909), the most famous Belgian diatomist whose Types du Synopsis Atlas (Van Heurck 1880–1885) was one of the first comprehensive illustrated diatom monographs in the world.

#### Distribution.

*Staurosirellavanheurckiana* has so far only been found in the type locality, most likely due to confusion with similar taxa such as *Staurosirellaovata*.

#### Ecology and associated diatom flora.

The type sample is one of the samples in the Van Heurck exsiccata set Types du Synopsis des Diatomées de Belgique (Van Heurck 1882–1885). The sample was labelled “*Fragilariamutabilis* (W.Sm.) Grun. *formae minores*” and was collected near Louvain (Leuven, prov. Vlaams Brabant, Belgium). The original vial of the sample also mentions the number “291” although it is unclear to what number this refers. Van Heurck (1885, p. 157) mentions that the Louvain sample was collected by P.G., which most likely stands for Père Vincent Gautier (1827–1903), who collected a large amount of samples that are now conserved in the Van Heurck collection in BR. The sample is dominated by *S.vanheurckiana* reaching more than 50% of all counted valves. Other, subdominant species include *Encyonemaventricosum* (C.Agardh) Grunow, *Melosiravarians* C.Agardh, *Nitzschialinearis* (C.Agardh) W.Smith, Ulnariacf.verhaegeniana Van de Vijver et al., *Gomphonemacapitatum* Ehrenberg, *Planothidiumlanceolatum* (Brébisson) Lange-Bertalot, *Amphoraovalis*, and *Meridioncirculare* (Gréville) C.Agardh, but they never reached more than 2% of the total flora. This diatom flora most likely points to medium to higher trophic levels, and organic pollution up to the β-α- mesosaprobic levels (Lange-Bertalot et al. 2017).

## ﻿Discussion

The combination of morphological features places all new species in the genus *Staurosirella*. The striae are composed of linear, apically aligned areolae, separated by thin vimines. Broad, often well-raised virgae separate the striae. Rimoportulae are absent. Apical pore field present, usually similar in shape and size on both apices. These features fit well with the description of the genus *Staurosirella* in Williams and Round (1988) and Morales and Manoylov (2006a).

Each of the new species can be distinguished from similar small-celled araphid taxa worldwide. Tables 1–4 highlight the different features of similar species.

*Staurosirellamarginostriata* shows most resemblance to both *S.canariensis* (Lange-Bertalot) E.Morales et al. and *S.krammeri* E.Morales et al. (Table 1). There are hardly any *Staurosirella* species with short and only marginal striae (Morales et al. 2010b). *Staurosirellakrammeri*, described from Oregon, USA, has an almost similar valve outline and comparable valve dimensions, but lacks apical pore fields often replaced by a marginal stria and has typically only one large, acute to spatulate spine per virga (Morales et al. 2010b, figs 33–35). Contrarily, *S.marginostriata* shows well-developed apical pore fields and irregular series of small marginal spines located on the virgae. *Staurosirellacanariensis* is much smaller (length up to maximum 7 µm), has a more elliptical, broadly rounded valve outline, and possesses small apical pore fields composed of only a few pores (Lange-Bertalot 1993). In LM, probably most confusion can be caused by *Pseudostaurosirabrevistriata*, despite the latter belonging to a completely different genus, which becomes clear when comparing the ultrastructure of both species. *Pseudostaurosirabrevistriata* forms long, chain-like colonies using well-developed linking spines, and possesses striae composed of one, transapically elongated areola. In LM, however, the marginal striae and the similar valve outline may result in an incorrect identification. Careful observation of the striae shows that they are more vaguely elongated in *S.marginostriata*, continuing into well-developed ghost striae whereas in *P.brevistriata*, the striae appear to be composed of one large, rounded, well-delimited areola (Morales et al. 2015).

**Table 1.** Comparison of valve features between *Staurosirellamarginostriata* sp. nov. and similar species.

	*Staurosirellamarginostriata* sp. nov.	*Staurosirellacanariensis*	*Staurosirellakrammeri*	*Pseudostaurosirabrevistriata*
reference	this study	Lange-Bertalot (1993)	Morales et al. (2010b)	Morales et al. (2015)
valve length (µm)	10–20	4–7	4–14	11–19
valve width (µm)	3.0–4.5	3–4	3–5	3.5–5.0
valve outline	lanceolate to rhombic-lanceolate	elliptic to elliptic-lanceolate	rhombical to elliptic	lanceolate to rhombic and elliptic in smaller valves
apices	weakly protracted, rostrate to not-protracted, cuneate in smaller specimens	not protracted, broadly rounded	not protracted, cuneate to broadly rounded	variable, ranging from narrowly or broadly rounded
apical pore field	large, present on both apices, 4–5 rows of large, rimmed pores	very small, present on both poles, 2 rows of a few small pores	absent	small, composed of a handful of areolae
spines	small, marginal, irregularly shaped, non-linking	small, marginal, irregularly shaped, non-linking	one per virga, large, spathulate, non-linking	spathulate linking spines
striae (in 10 µm)	14–15	12–15	12–14	13–14
stria structure	composed of a few, apically linear areolae	composed of a very few, apically linear areolae	composed of a few, apically linear areolae	composed of one large, transapical areola

*Staurosirellabinodiformis* shows some resemblance to *Staurosirabinodis* Ehrenberg, *Staurosirellaconfusa* E.Morales, and *Staurosirellaoldenburgiana* (Hustedt) E.Morales (Table 2). The type material of *S.binodis* from Santa Fiore, Italy has recently been investigated (Van de Vijver, unpublished results). *Staurosirabinodis* presents the typical structure of the genus *Staurosira*: frustules forming long, ribbon-like colonies, each frustule connected to the next using large, spathulate linking spines; large apical pore fields composed of numerous rows of small, rounded pores, long striae composed of rounded, small areolae and numerous girdle bands. In LM, the valve outline, linear with a clear central constriction, may cause some confusion with *S.binodiformis*, but the lack of colonies and the more narrow valves (max. 3 µm vs 4–6 µm in *S.binodis*) of the latter, limit the confusion. The most similar species appears to be *S.oldenburgiana*, a species described by Hustedt in 1959 from the Oldenburg Canal (Lower Saxony, Germany), although the valves are a little bit broader (up to 4 µm), with more elongated, narrower apices. The most distinct difference is the number, shape and structure of the marginal spines (Hustedt 1959; Morales 2005). Whereas *S.binodiformis* has a continuous series of very small, blunt spines, usually located on each side of the striae, *S.oldenburgiana* possesses only one, long, acute marginal spine located in the middle of the virgae (Morales 2005, Van de Vijver unpubl. res.). Hustedt (1959, p. 29) most likely had observed this taxon already as he mentioned in the discussion of his new taxon *Fragilariaoldenburgiana*, “*Ähnliche Erscheinungsformen in Island [Similar forms in Iceland*]”. Foged (1974, plate 3, fig. 3) illustrated one valve as Fragilariaconstruensvar.binodis (Ehrenberg) Grunow, but the depicted valve clearly shows *S.binodis* and not the new *Staurosirellabinodiformis*. *Staurosirellaconfusa*, finally, has a more lanceolate valve outline with usually convex margins and elongated, narrow rostrate apices, lacking in *S.binodiformis*. In Turkiye, a similar taxon was observed showing narrower valves (valve width max. 2.5 µm) giving that taxon a more slender, smaller outlook (C. Solak, pers. comm.). It is unclear whether those Turkish populations also belong to *S.binodiformis*. They were observed in a typical karstic environment, different from the Icelandic habitat.

**Table 2.** Comparison of valve features between *Staurosirellabinodiformis* sp. nov. and similar species.

	*Staurosirellabinodiformis* sp. nov.	*Staurosirellaoldenburgiana*	*Staurosirellaconfusa*	*Staurosirabinodis*
reference	this study	Hustedt (1959) + unpublished results	Morales (2005)	Van de Vijver, unpublished results
valve length (µm)	6–21	10–20	11–20	15–21
valve width (µm)	3.0–3.5	3–4	3.5–4.5	4–6
valve outline	isopolar, linear with constricted valve middle in longer valves, lanceolate to elliptic lanceolate with convex margins in smaller specimens	isopolar, narrowly linear with weakly constricted margins	weakly heteropolar, lanceolate	linear with distinctly constricted central part
apices	protracted, rostrate to subcapitate (longer valves) to not protracted, broadly rounded in smaller specimens	protracted, very elongated, rostrate to subcapitate	protracted, elongated rostrate	protracted, rostrate to subcapitate
apical pore field	large, present on both apices, 2–3 rows of large, rimmed pores	large but compact, 4 short rows of large, rimmed pores	very large, at both apices, composed of up to 7 long rows of large pores	large, at both apices, composed of several rows of very small pores
spines	small, marginal, irregularly shaped, non-linking	small, marginal, acute, one per virga	large, spathulate, linking spines	spathulate linking spines
striae (in 10 µm)	14–15	ca. 13	ca. 11	15–16
stria structure	radiate to more strongly radiate at the apices, composed of linear, apically oriented areolae	almost parallel, composed of linear, apically oriented areolae	broad striae, composed of long, apically elongated areolae	long, parallel to weakly radiate, composed of small, rounded areolae

*Staurosirellastoksiana* bears some similarity to a large number of European and North-American *Staurosirella* species (Table 3). The most similar species include *S.ovata* E.Morales and *S.martyi* (Héribaud) E.Morales & Manoylov, not in the least because of their heteropolar, often ovoid valve outline. Both, however, entirely lack spines, and have a more heteropolar valve outline with a clear difference between the very broadly rounded headpole and the more acutely rounded footpole (Morales and Manoylov 2006b, Van de Vijver et al. in press). *Staurosirellamartyi* (syn. *Staurosirelladubia* (Grunow) E.Morales & Manoylov) usually has larger valves with a valve width up to 10 µm, whereas in *S.stoksiana*, the valve width rarely exceeds 5.5 µm. Larger valves of *S.martyi* also show a clear constriction just below the headpole, a feature lacking in *S.stoksiana.Staurosirellaneopinnata* E.Morales et al. has typically parallel margins, isopolar valves, smaller apical pore fields and more regularly placed marginal spines (Morales et al. 2019) and *S.coutelasiana* Van de Vijver has longer, more slender valves, a less heteropolar valve outline, and a higher stria density (9–11 vs 8–9 in 10 µm (Van de Vijver 2022). The newly described *Staurosirellavanheurckiana* differs in a lower valve width (3.0–3.5 µm), a slightly wider sternum, a more clear heteropolar, ovoid valve outline with a broadly rounded head pole throughout its entire cell diminution series, the complete absence of marginal spines despite the analysis of a large population, and a different apical pore field structure with parallel rows of areolae along the apical axis (contrary to the transapical rows in *S.stoksiana*).

**Table 3.** Comparison of valve features between *Staurosirellastoksiana* sp. nov., *S.vanheurckiana* sp. nov. and similar species.

	*Staurosirellastoksiana* sp. nov.	*Staurosirellavanheurckiana* sp. nov.	*Staurosirellaneopinnata*	*Staurosirellaovata*	*Staurosirellacoutelasiana*	*Staurosirellamartyi*
reference	this study	this study	Morales et al. (2019)	Morales and Manoylov (2006b)	Van de Vijver (2022)	Van de Vijver et al. (unpublished results)
valve length (µm)	6–21	8–22	4–25	6.5–38	15–35	9–38
valve width (µm)	4.5–5.5	3.0–3.5	4–5	3.5–7	5.0–5.5	5–10
valve outline	heteropolar, larger valves ovoid, smaller valves more elliptical, convex margins, gradually narrowing	heteropolar, lanceolate to elliptic-lanceolate, smaller specimens entirely ovoid	usually isopolar with parallel margins	typically heteropolar, ovoid	isopolar to occasionally very slightly heteropolar, linear-lanceolate in larger specimens to lanceolate, occasionally elliptic-lanceolate in smaller valves	heteropolar, larger valves with clear constriction between valve middle and headpole, smaller valves ovoid in shape
apices	not protracted, broadly rounded	not protracted, head pole broadly rounded, foot pole acutely rounded	not protracted, broadly rounded	headpole broadly rounded, footpole more acutely rounded	broadly rounded, not protracted	headpole broadly rounded, footpole more acute
apical pore field	present on both apices, larger pore field at footpole composed of up to 8 rows of very small pores, at headpole smaller and located on depression	pore field at head pole small, located on small depression, pore field at foot pole large, composed of a large number of parallel rows of very small rounded pores	very small, on both apices, composed of a handful of small pores	present at both apices, but more developed at the footpole.	present on both apices, similar in size and shape, composed of a handful of larger pores	at footpole very large, composed of more than 8 long rows of small pores, at headpole restricted to a compact group of a few small pores
spines	small, marginal, irregularly shaped, non-linking, occasionally valves without spines observed	Absent	small, irregularly shaped, 2–3 per virga, non linking	absent	marginal on the virgae, irregularly shaped (acute to spatulate), usually one or two per virga	absent
striae (in 10 µm)	8–9	8–10	8–9.5	6–9	9–11	6–7
stria structure	alternating, almost parallel becoming very slightly radiate towards the apices	alternating, almost parallel to very slightly radiate throughout	parallel almost throughout the entire valve	parallel to slightly radial toward the poles	alternating at both sides of the sternum, parallel to weakly radiate near the valve middle, becoming distinctly more radiate towards the apices	parallel in the middle becoming gradually weakly radiate towards the apices

There are a few *Staurosirella* species that show some similarity to *S.jonssoniana*, based on the lanceolate valve outline with shortly protracted, rostrate apices (Table 4). The most similar is *S.acidophila* Almeida et al., described from southeastern Brazil. The species has a more elliptical-lanceolate central part with clearly convex margins and well protracted, elongated rostrate apices. The valves are always larger (width 4.5–7 µm vs 3.0–3.5 µm), have distinct apical pore fields (contrary to *S.jonssoniana* lacking apical pore fields), and with a different spine density (at least 2 per virga) and structure (lack of the pit-like depressions) (Almeida et al. 2015). *Staurosirellaconfusa* is also slightly larger (3.5–4.5 µm) with larger apical pore fields and at least 2 spines per virgae, each not located in a depression (Morales 2005). There are hardly any *Staurosirella* species lacking an apical pore field and most of these form long colonies such as *S.mutabilis* (W.Smith) E.Morales & Van de Vijver and *S.lapponica* (Grunow) D.M.Williams & Round (Van de Vijver et al. 2022). In LM, confusion may also arise with members of the genus *Punctastriata* D.M.Williams & Round, described in 1988 (Williams and Round 1988). Especially *P.linearis* D.M.Williams & Round has a more or less similar valve outline, although this species is always typically heteropolar with a more broadly rounded head pole and a more acute foot pole. The ultrastructure is entirely different with the typical *Punctastriata* feature of striae composed of several rows of small, rounded areolae. *Punctastriatalinearis* also possesses one apical pore field on the foot pole and a clear depressed head pole, although these features are difficult to see in LM (Wetzel and Ector 2021). The larger *Staurosirellasubcapitata* E.Morales differs in the presence of apical pore fields and a lower stria density (7–9 in 10 µm vs 12–13 in 10 µm) (Morales and Manoylov 2006b).

**Table 4.** Comparison of valve features between *Staurosirellajonssonii* sp. nov. and similar species.

	*Staurosirellajonssonii* sp. nov.	*Staurosirellaconfusa*	*Staurosirellaacidophila*	*Staurosirellasubcapitata*	*Punctastriatalinearis*
reference	this study	Morales (2005)	Almeida et al. (2015)	Morales and Manoylov (2006b)	Wetzel and Ector (2021)
valve length (µm)	6–15	11–20	11–20	8–27	12–20
valve width (µm)	3.0–3.5	3.5–4.5	4.5–7	4.0–5.5	1.5–3
valve outline	isopolar to weakly heteropolar, lanceolate, with convex margins	weakly heteropolar, lanceolate	isopolar to slightly heteropolar, lanceolate to rhombic-lanceolate	isopolar, lanceolate	weakly heteropolar, lanceolate to elliptical in smaller valves
apices	narrowly protracted, rostrate	protracted, elongated rostrate	acuminate to rostrate rounded	acutely rounded, rostrate to subrostrate	shortly protracted, rostrate
apical pore field	absent	very large, at both apices, composed of up to 7 long rows of large pores	present at both apices, composed of small, rimmed pores	present on both apices, similar in size and shape, composed of several rows of small pores	one apical pore field present at foot pole, depression lacking pore field at head pole
spines	marginal, one per virga, each located in a shallow, pit-like depression	large, spathulate, linking spines	small, 2-3 located in costae valve, sometimes in double rows	present on the virgae, occasionally absent	irregular row of short, acute spines, placed on virgae and striae
striae (in 10 µm)	12–13	ca. 11	8–9	7–9	10–13
stria structure	alternating, almost parallel to very slightly radiate in the middle, more strongly radiate towards the apices	broad striae, composed of long, apically elongated areolae	alternating, slightly radiate	alternating, parallel, becoming radiate at the apices	parallel, composed of 4-6 rows of small, rounded areolae

Finally, *Staurosirellavanheurckii* shows some resemblance to *S.stoksiana* but as highlighted here above, both can be separated based on valve outline, valve width, absence/presence of marginal spines and the structure of the apical pore fields (see Table 3). *Staurosirellaovata*, also lacking spines, is much wider (3.5–7.0 µm, i.e. double the valve width of *S.vanheurckiana*) and has a different apical pore field structure (Morales and Manoylov 2006b). The same applies for *S.martyi* that has a different valve outline, especially in the longer valves and a higher valve width (5–10 µm). Both *S.ovata* (6–9 in 10 µm) and *S.martyi* (6–7 in 10 µm) have a lower stria density compared to *S.vanheurckiana* (8–10 in 10 µm).

## ﻿Conclusions

The description of these five new European *Staurosirella* species is the result of the continuous revision of the genus *Staurosirella* following the careful analysis of the historic type material. Catch-all species such as *S.pinnata* (now *S.neopinnata*), *S.mutabilis* and *S.martyi* revealed a much higher diversity in the genus *Staurosirella* than previously understood. It is clear that more species will be discovered in the near future, when more populations, in the past identified as S. (neo) pinnata will be investigated. Morphological criteria such as valve outline and valve dimensions need to be completed with a better analysis of other structures such as the shape, size and structure of the apical pore fields, the structure and density of the marginal spines, the presence (or absence) of a head pole depression and the structure of the striae in combination with the virgae.

## Supplementary Material

XML Treatment for Staurosirella marginostriata

XML Treatment for Staurosirella binodiformis

XML Treatment for Staurosirella stoksiana

XML Treatment for Staurosirella jonssoniana

XML Treatment for Staurosirella vanheurckiana

## ﻿Additional information

### Author ORCIDs

Bart Van de Vijver https://orcid.org/0000-0002-6244-1886

Iris Hansen https://orcid.org/0009-0007-3315-3344

Myriam de Haan https://orcid.org/0000-0003-1868-1265

### Data availability

All of the data that support the findings of this study are available in the main text.
